# Over prescription of antibiotics in children with acute upper respiratory tract infections: A study on the knowledge, attitude and practices of non-specialized physicians in Egypt

**DOI:** 10.1371/journal.pone.0277308

**Published:** 2022-11-03

**Authors:** Mariam Taher Amin, Mahmoud Attia Abd El Aty, Sabra Mohamed Ahmed, Ghada Omar Elsedfy, Ebtisam Shawky Hassanin, Amira Fathy El-Gazzar

**Affiliations:** 1 Faculty of Medicine, Public Health and Community Medicine Department, Assiut University, Assiut, Egypt; 2 Faculty of Medicine, Department of Pediatrics, Children’s Hospital, Assiut University, Assiut, Egypt; 3 Ministry of Health and Population, Assiut, Egypt; 4 Badr University, Cairo, Egypt; Kohat University of Science and Technology, PAKISTAN

## Abstract

**Background:**

Antimicrobial resistance (AMR) is currently one of the global public health threats. Increased antibiotic consumption in humans, animals, and agriculture has contributed directly to the spread of AMR. Upper respiratory tract infections (URIs) are one of the most common conditions treated by antibiotics, even if unnecessary as in cases of viral infections and self-limited conditions which represent the most cases of URIs. Investigating physicians’ knowledge, attitudes, and practice regarding antibiotic prescriptions in children with acute URIs may reflect the problem of antibiotic over prescription. This study aims to assess the problem in our community and provide information for further planning of appropriate interventions to optimize antibiotic prescriptions.

**Methods:**

This is a cross-sectional study for all non-specialized physicians dealing with acute upper respiratory tract infections (URIs) in pediatrics sittings in Assiut district, Egypt. We used a self-administered questionnaire to assess physicians’ knowledge, attitudes, and practice. In addition, four clinical vignettes addressing different URI scenarios were included in the questionnaire to assess the patterns of antibiotic prescriptions in common cases.

**Results:**

Our study included 153 physicians whose mean age was 32.2 ± 8.7, most of whom were pediatric residents in different health institutes in Assiut district. They had good knowledge as out of the 17 knowledge questions,the mean number of correct answers was 12.4 ± 2.9. Regarding their attitudes, mean attitude scores for inappropriate antibiotic prescribing were low. However, of those scores, the responsibility of others had the highest score (3.8 ± 0.61). Prescribing practice in special conditions of URIs showed that 80% of participants prescribed antibiotics if fever continued for more than five days and 61.4% if the child had a yellowish or greenish nasal discharge. Among 612 clinical vignettes, 326 contained antibiotic prescriptions (53.3%), and appropriate antibiotic prescriptions represented only 8.3% overall.

**Conclusions:**

Physicians dealing with acute URIs in outpatients’ clinics in the Assiut district have good knowledge about antibiotic use and resistance and demonstrate a good attitude toward appropriate antibiotic use. Although the percentage of inappropriate prescriptions in clinical vignettes in high, more research is required to investigate the factors of antibiotic inappropriate prescribing practice and non-adherence to guidelines. Also, it is essential to set up a national antibiotic stewardship program to improve antibiotic prescribing and contain antimicrobial resistance problems.

## Background

One of the most important medical advancements of the 20th century was the development of antibiotics, which represent the largest class of medications. The development of antibiotics has benefited human society in the fight against microorganisms and saving millions of lives [1,2]. However, microorganisms such as bacteria are living organisms and their main objective is to survive and spread so when facing threats, they developed mechanisms to insure their survival. Although the antimicrobial resistance is a natural response and usually occurred by genetic modifications, but irrational human use of antibiotics facilitates and accelerate this process [3,4]. The human factors that accelerated emergence of AMR includes, antibiotic overuse and abuse, incorrect diagnosis and improper antibiotic prescribing, self-medication, bad healthcare environments, poor personal hygiene, and widespread agricultural use [1].

Antimicrobial resistance is a significant concern for global public health and a major threat to global health security, with an increasing number of resistant bacteria strains identified annually in both human and animal populations in developed and developing countries [5]. Based on a recent study of the global burden of antimicrobial resistance, it is estimated that global all-age death rate attributable to resistance was 16.4 /100,000. At the regional level, the death rate was highest in western sub-Saharan Africa (27·3 /100,000) and lowest in Australasia (6·5 /100,000). In our region -North Africa and Middle East- the death rate attributable to resistance estimated to be 11.2 /100,000. Another rate estimated in this study was disability-adjusted life-years (DALYs), globally the DALYs rate attributable to resistance was 618·7/100,000 and in North Africa and Middle East was 429/100,000. The study also reported that, AMR all-age death rates were highest in LMICs, making AMR not only a major health problem globally but a particularly serious problem for some of the poorest countries in the world [6].

Policymakers, health organizations, and research institutes have advocated for greater control over the distribution and use of antibiotics in society, with a particular focus on antibiotic prescribers and dispensers [7]. Despite decades-long efforts to encourage the "rational use of drugs," such as the World Health Organization’s International Network for the Rational Use of Drugs program (WHO’s-INRUD), antibiotic use appears to be on the rise [8]. The joint effort of the WHO, World Organization for Animal Health (WOAH), and (FAO) Food and Agriculture Organization to define a Global Action Plan (GAP) led to five central objectives. The first prioritized raising awareness about antimicrobial resistance (AMR), and the fourth was optimizing antimicrobial use in humans and animals [7].

Antibiotics are among the most frequently recommended drugs in pediatric treatment worldwide. Antibiotic overuse is a significant public health issue and crucial in establishing antimicrobial resistance [9,10]. Besides the development of resistance, using excessive antibiotics in early life has been associated with microbiome alteration. Microbiome research and the recognition of the vital role of microbiota in human health have revealed the undesirable effects of using antibiotics, particularly, during the early years of life. Antibiotic use has evidenced to alter both adult and infant microbiome so that a short course of antibiotic can leave a long impact of 6 months on microbiota composition [11]. The microbial imbalance can cause serious long -term negative impact on health such as autoimmune diseases, metabolic diseases, and malnutrition [12–15]. Moreover, there are concerns regarding the abuse of broad-spectrum antibiotics such as Amoxicillin/clavulanate, even for diseases where their usage may be justifiable [16,17].

Upper respiratory tract infections (URIs) are among the leading causes of childhood morbidity. Additionally, it is one of the most common reasons for children seeking health care in outpatient settings, posing an enormous burden on society and the health care system [1]. Therefore, examining prescriptions for childhood URIs is a commonly accepted strategy to evaluate the rationality of antibiotic use [18].

In Egypt, the rationality of antibiotic use in such cases is not well studied. Besides, a few scattered studies were conducted in Egypt on antibiotic prescribing practice among physicians. In 2020, a cross-sectional study was conducted by inviting physicians to evaluate their knowledge, attitudes, and practice regarding antibiotics. In this study, 500 physicians were recruited from across Egypt’s governorates. The study revealed that Egyptian prescribers have considerable knowledge of antibiotics. However, there are low rates of good attitudes and proper practices toward the problem of AMR [19]. Furthermore, in Minya district -one of the upper Egypt governorates- a study has been conducted to determine attitudes and practices about antibiotic prescribing for outpatient acute respiratory infections. According to this study, there is a significant rate of unjustified outpatient antibiotic prescribing and dispensing [20]. Therefore, we conducted this study to investigate physicians’ knowledge, attitudes, and practice regarding antibiotic prescribing in children with acute upper respiratory tract infections, aiming to assess the problem in our community and provide information for further planning appropriate interventions to optimize antibiotic prescriptions in such cases.

## Methods

### Study design and setting

This cross-sectional study was conducted in Assiut district and targeted physicians dealing with outpatient pediatric cases. The current study is compliant with the "Strengthening the Reporting of Observational Studies in Epidemiology Statement" (STROBE) checklist [21].

Assiut district is the central district of Assiut Governorate, one of the upper Egypt governorates, lying 375 km south of Cairo. Based on the last census in 2017, the population of Assiut district was 974,993 [22].

Outpatient clinics dealing with pediatric cases in Assiut district are distributed among the following centers and hospitals: rural and urban primary health units (14 rural units and 19 urban units); Ministry of Health hospitals (3 hospitals, Al-Iman General Hospital, Al-Shamla Hospital, and Women and Children’s Hospital); and Assiut University Hospitals (Assiut University Children’s Hospital and ENT clinics from the main hospital).

### Study population

Inclusion criteria for this study were any non-specialized physicians dealing with outpatients’ pediatric cases having acute URIs at study sites (GPs, pediatrics, or ENT residents). Physicians who did not work at outpatient clinics and those with a postgraduate degree were excluded.

As it is exploratory study and the primary aim was to study the situation of antibiotic prescribing practice, total coverage sampling technique was adopted and all eligible physicians in study area were invited to complete the questionnaire.

According to data from the directorate of the Ministry of Health in Assiut and Assiut University Hospital, there were 80 GPs at urban and rural primary health care (PHC) units, 35 pediatric residents, and 8 ENTs at MoHP hospitals. At Assiut University Hospitals, there were 50 pediatric and 9 ENT residents. Thus, the eligible physicians were estimated to be 182 physicians.

### Study instrument

Data of the present study were collected through a self-administered questionnaire developed and designed by the investigator. For this, a search of primary studies and reviews published in English language was conducted on the PubMed, Medline and Cochrane databases from January 2010 to December 2021 using the following search term strategy: “antimicrobial resistance” OR “antibiotic prescription” AND “perception” OR “attitudes” OR “knowledge” AND “upper respiratory tract infections” AND “pediatrics” OR “Children”. Then evaluation of retrieved papers and tools was performed, and relevant tools was chosen based on our study objectives [19,23–25]. Also, clinical vignettes were developed to assess the antibiotic prescribing behavior of the participants.

The questionnaire consisted of four parts. First, socio-demographic and professional characteristics; this part includes personal and demographic factors related to physicians. Also, questions about their postgraduate studies, continuous medical education (CME), and factors influencing their prescriptions were included. The second part is knowledge questions; this part includes 17 items and responses selected as yes, no, or do not know. The questions were divided into two groups: knowledge of antibiotics to test the physicians’ background on antibiotics and their use (8 items), and knowledge of antibiotic resistance to test physicians’ knowledge of antibiotic resistance and its causes (9 items). The third part includes attitude questions; 27 items assessed five attitudes aspects according to the Teixeira Antibiotic Prescribing Behavioral Model [23]:

- Ignorance: a lack of concern for antibiotic resistance resulting from over-prescribing antibiotics (7 items).
- Responsibility of others: a belief that others (patients, governments, and other professionals) are responsible for the problem of antibiotic resistance (4 items).
- Indifference: a lack of motivation to change antibiotic prescribing practices (3 items).
- Complacency: prescribing antibiotics to satisfy patient demands and expectations (8 items).
- Fear: prescribing antibiotics for fear of losing patients or losing in potential disputes with patients (5 items).

The attitude questions were designed according to the five-point Likert scale, having options of "very disagree," "disagree," "neutral," "agree," and "very agree" for the first three aspects (ignorance, responsibility of others, and indifference). However, for the last two aspects (complacency and fear), the scale having options of "never," "rarely," "sometimes," "often," and "always" was used. Cronbach’s alpha was used to calculate the reliability of attitude scales. The internal consistency (alpha) for all attitude scales was 0.80, indicating good consistency.

The fourth part includes two types of questions to assess physician practice. First, questions about their choice in specific conditions of upper respiratory tract infections and whether they will prescribe antibiotics "always," "sometimes," or "never" in such cases. Second, they were asked to assess four different clinical vignettes; for each case 7 items were evaluated, they were asked about the suggested diagnosis, if the antibiotic was needed, and if yes, what was the type and duration. They were also asked if they would prescribe injectable or combined antibiotics and if they will ask parents to comeback for follow-up.

Each case was assessed to see if it had an inappropriate antibiotic prescription or not based on WHO-aware guidelines, Nelson’s Pediatric Antimicrobial Therapy recommendations, and Assiut University Hospitals antibiotic guidelines [26–28]. In this study, the operational definition used for inappropriate prescription was the WHO inappropriate prescription definition. According to this definition, the he prescription is considered inappropriate according to the WHO definition in the following cases: a) if not indicated, b) if indicated but the wrong type and/or duration of the antibiotic has been prescribed, and c) use of injectable antibiotics or combination of antibiotics if there is no actual indication for this [29]. The clinical vignettes and recommended treatment are presented in Table 1.

**Table 1:** Recommended antibiotics for clinical vignettes.

Case	Clinical Vignette	Suggested Diagnosis	Recommended Antibiotic type and duration
1	A 5-year-old girl developed a high-grade fever for 2 days (39°C) and a sore throat. Two days before the fever, she had nasal flu and cough. On examination, the pharynx was congested; otherwise, no other remarkable findings.	Viral acute URIs	No antibiotic is needed
2	A playful one-and-half-year-old child had a runny nose for two days, followed by a fever (38°C). He had a dry cough and, as per his mother, could not sleep because of his stuffy nose.	Common Cold	No antibiotic is needed
3	A 4-year-old child complained of ear pain. He had a temperature of 38.9°C. He had a cold for several days but was eating well, and his activity was normal.	Acute Otitis media	Amoxicillin OR Amoxicillin + Clavulanic acid for *5 days*
4	A 9-year-old boy developed a high-grade fever of 3 days (40°C). He complained that he could not eat because of throat pain. On examination, the pharynx was congested with excaudate on the tonsils. Also, there were palpable cervical lymph nodes.	Acute Follicular Tonsilitis	Amoxicillin OR Phenoxymethyl Penicillin orally *for 10 days* OR Benzathine Penicillin IM injection as a single dose.

#### Validity study questionnaire

Content validity of the instrument was evaluated in two different stages in accordance with published guidelines [30]. First is the development stage, which involves reviewing the literature to build the questionnaire and determine items. Second is the judgment stage, in which the professional opinion of pediatrics and public health experts is evaluated. This stage aimed to pre-test the questionnaire and assess the accuracy, clinical terminology, completeness, and meaning of all the statements for content validity. Also, face validity was assessed, which includes an evaluation of the grammar, clarity of language, organization, appropriateness, and logical sequence of the statements [31].

### Ethics approval and consent to participate

The study protocol was reviewed and approved by the Institutional Review Board of Faculty of Medicine, Assiut University (IRB no.: 17200370) and registered on clinicaltrials.gov (NCT04127682). A formal permission letter was obtained from the Directorate of the Ministry of Health in Assiut.

The questionnaire cover letter informed participants about the purpose of the study, ensured all information would remain confidential and informed them that consent to participate was implied upon their agreement to participate and by signing of cover letter and completing the questionnaire.

### Pilot study

A pilot study was carried out on 20 physicians before data collection, and their results were not included in the analysis. The pilot aimed to test the reliability of the questionnaire by the test-retest method. It also aimed to estimate the required time for implementing the questionnaire, which was 20–30 minutes. Therefore, an interval of 2 to 4 weeks was allowed to elapse between the first and second administration of the questionnaire.

### Data collection

Data were collected from September 2021 to February 2022 as this is the time of year with increased URIs (fall and winter seasons). Two well-trained research assistants from the research office of the Directorate of the Ministry of Health in Assiut helped the researcher to distribute the questionnaires in MoHP hospitals and PHC units and to watch, keep understanding and completeness of data, and answer all the questions asked by the physicians to ensure the accuracy and completeness of data. Aim of the study was explained to physicians and instructions to complete the questionnaire has been given.

### Statistics

Data were entered in Microsoft^®^ Excel 2010 and then transferred to Stata/IC^®^ 16.1 for further data management and analysis. Incomplete questionnaires with more than 50% missing data or incomplete responses to clinical vignettes were excluded from the analysis. Graphic presentations were conducted using Microsoft^®^ Excel 2010.

The frequency and percentage of correct answers were calculated for the knowledge score. For each aspect, the mean score of each participant was calculated and ranged from 1 to 5; the higher the score, the more inappropriate the prescription attitude. Quantitative data were presented as mean ± SD or median (min-max), while categorical data were presented as frequencies and percentages. For comparison of knowledge score according to post-graduate degree enrolment status and between inappropriate prescriptions and appropriate or no prescription cases; students’ t-test was used. To examine the correlation between knowledge and attitude scores Spearman’s correlation was performed. In all statistical tests p-value <0.05 was considered statistically significant.

## Results

**Fig 1:**
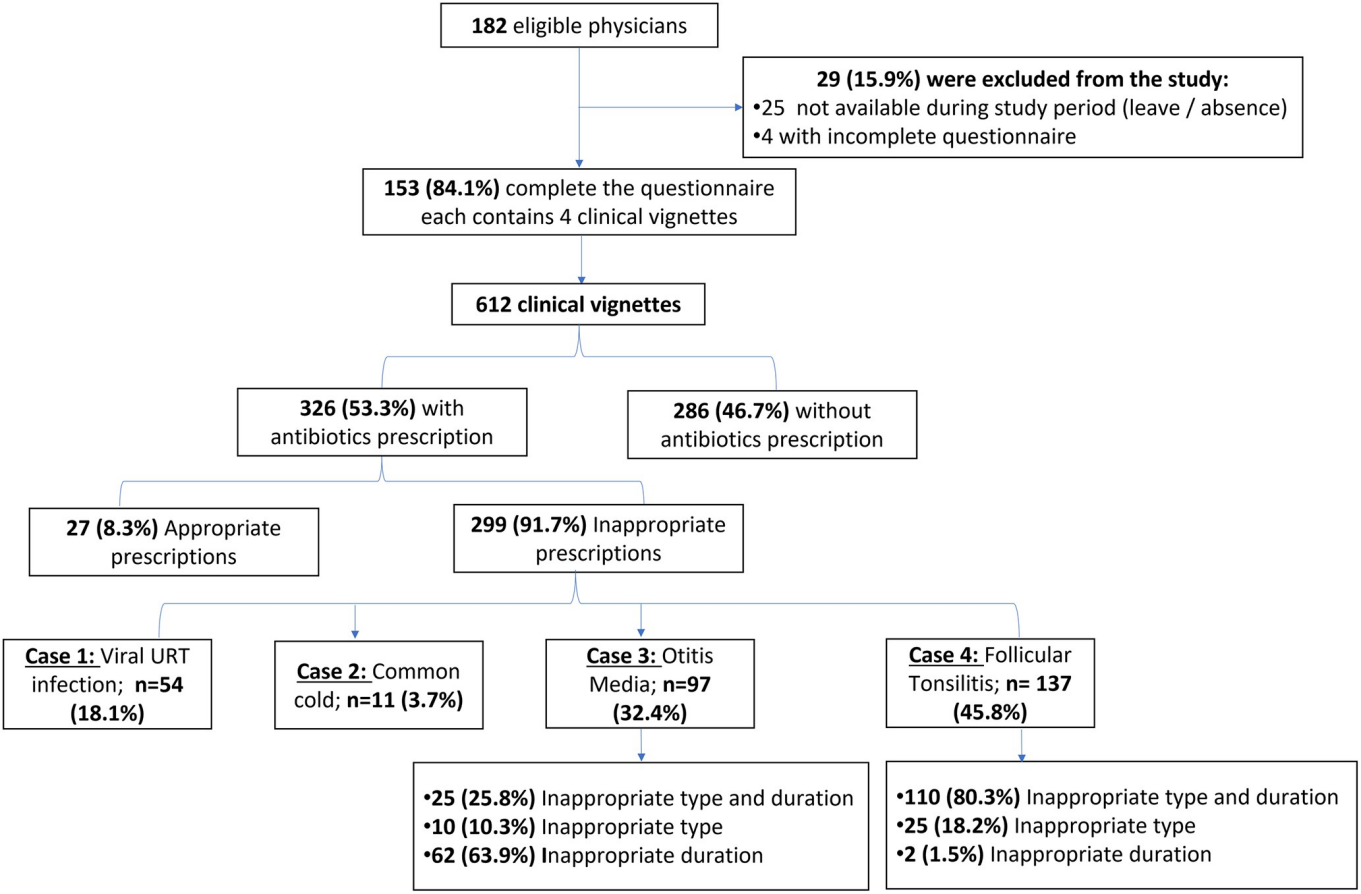
Flow diagram of the study illustrating responses to clinical vignettes.

Of 182 eligible physicians, 153 (84.1%) completed the questionnaire and were subjected to the full analysis. Twenty-five physicians were excluded for not being available during data collection time, and four questionnaires were excluded due to incomplete data (Fig 1).

The mean age of respondents was 32.2 ± 8.7, and 81.7% were less than 35 years old. The median duration for practice since graduation was four years, and junior doctors in practice for less than one year represented 26% of participants. Females represented 57.5% of respondents. Most study participants work in the university hospital (39.2%); pediatric residents represented the most significant proportion (64.1%), while GPs represented 23.5%. Twenty-four percent of participants did not enroll in any postgraduate degree, while 53.6% enrolled in a pediatric master’s degree. Nearly half of the participants received integrated management of childhood illness (IMCI) training, and 43.1% used IMCI guidelines in their practice. Last year, only 28% of the participants attended continuous medical education events addressing antimicrobial resistance (Table 2).

**Table 2:** Socio-demographic and professional characteristics of the study participants (n = 153).

Variable	Frequency	%
**Age (years)**		
24–35	125	81.7
> 35	28	18.3
***Mean ± SD***	32.2 ± 8.7	
**Years of Practice**		
≤ 1	40	26.1
> 1–5	60	39.2
> 5	53	34.6
***Median (Min–Max)***	4 (4 months-35 years)	
**Gender**		
Male	65	42.5
Female	88	57.5
**Place of work**		
University Hospital	60	39.2
Ministry of Health Hospital	44	28.8
Urban PHC unit	37	24.2
Rural PHC unit	12	7.4
**Job title**		
GP	36	23.5
Pediatric Resident	98	64.1
ENT Resident	12	7.8
Family Physician	7	4.6
**Postgraduate degree enrollment**		
No	37	24.2
Master of pediatrics	82	53.6
Master of ENT	13	8.5
Master of family medicine	2	1.3
Egyptian fellowship program ^**a**^	19	12.4
**IMCI Attendance**	74	48.4
**Use of IMCI in practice**	66	43.1
**Attendance of CME address AMR last year**	43	28.1

PHC: Primary health care, GP: General practitioner, ENT: Ear, nose & throat, IMCI: integrated management of childhood illness, CME: Continuous medical education, AMR: Antimicrobial resistance.

^**a**^ Either family medicine or pediatrics fellowship program.

**Fig 2:**
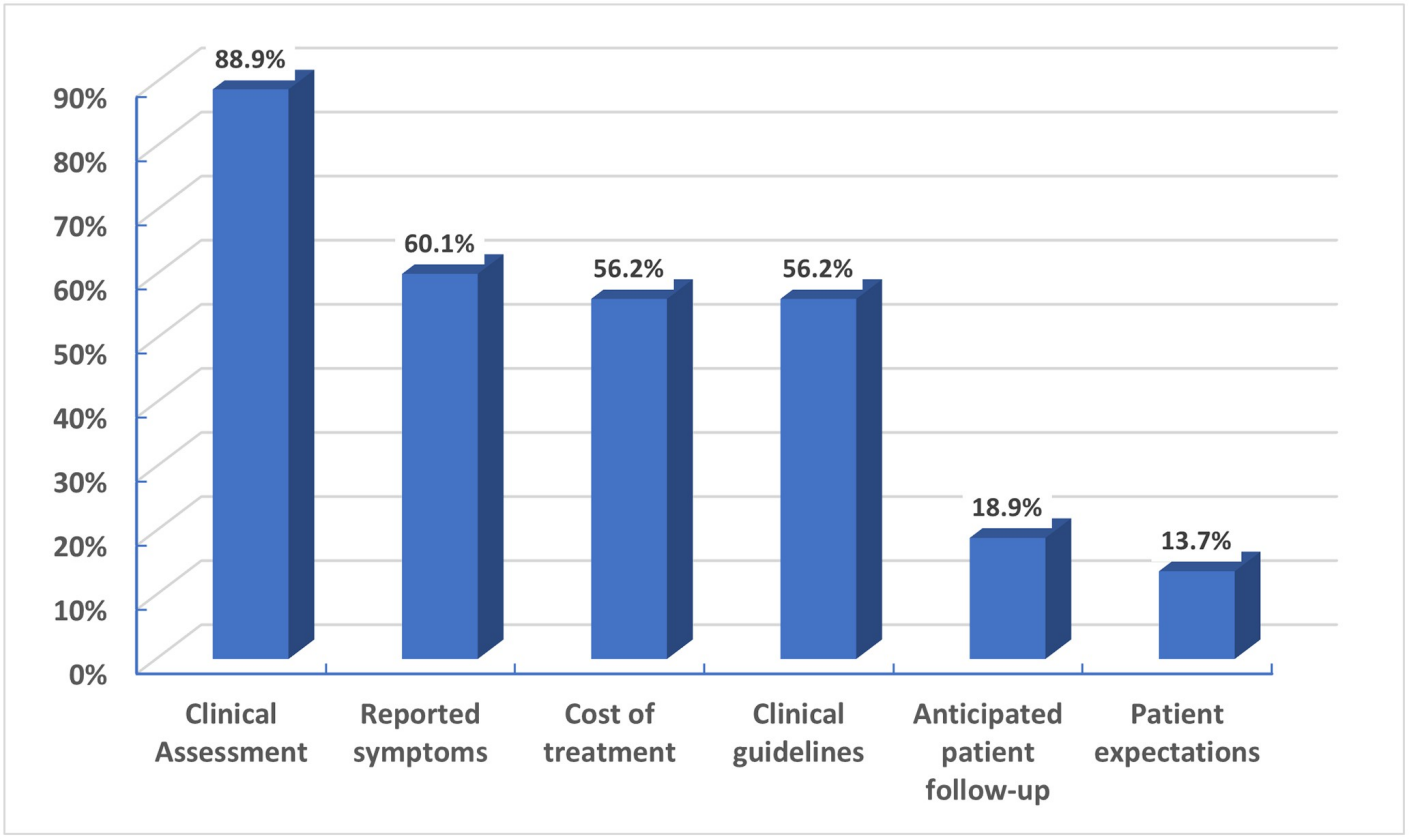
Factors affecting antibiotic prescribing as reported by participants.

Nearly eighty-nine percent of participants reported that they rely on their clinical assessment for prescribing antibiotics in cases of upper respiratory tract infections, followed by reported symptoms by the patients or their parents (60%). In contrast, patient expectations were reported to have a minor influence on the decision of antibiotic prescribing in those cases (Fig 2).

As regards their knowledge about antibiotics use and resistance, the participants have adequate knowledge as the mean number of correct answers for 17 knowledge questions was 12.4 ± 2.9. The most incorrectly answered questions in the knowledge of antibiotics were second and third, as 61.4% reported that broad-spectrum antibiotics are preferred in treating URT infections, and nearly half of them believe that antibiotics are an effective drug for fever. At the same time, the most incorrectly answered questions in antibiotic-resistant knowledge were the fourth and fifth questions, as 77.8% of participants did not know that antibiotic resistance can spread from animals to humans, and 54.9% did not know that antibiotic resistance can spread from person to person (Table 3). There was no statistically significant difference in mean score between those enrolled in postgraduate degrees and those didn’t enroll in any degree as mean score of postgraduate degree participants was 12.26 ± 3.1 and for others was 12.86 ± 2.0 (p-value = 0.264).

**Table 3:** Knowledge about antibiotic use and antibiotic resistance among participants.

Statement (correct answer)	Correct answers n (%)	Incorrect/ uncertain answers n (%)
**Knowledge of antibiotics:**		
Antibiotics are safe drugs, so that they can be used without prescriptions. ***(No)***	152 (99.4%)	1 (0.6%)
Broad spectrum antibiotics are more preferred for treating upper respiratory tract infections. ***(No)***	59 (38.6%)	94 (61.4%)
Antibiotics are not effective drugs for the treatment of fever. ***(Yes)***	76 (49.7%)	77 (50.3%)
There is no difference between co-amoxiclav (amoxiclav) and amoxicillin. ***(No)***	104 (68.0%)	49 (32.0%)
Antibiotics are not supposed to kill all bacteria in the body ***(Yes)***	90 (58.8%)	63 (41.2%)
The body can usually fight mild infections on its own without antibiotics ***(Yes)***	119 (77.8%)	34(3.9%)
Susceptibility tests help determine the likelihood that a particular antibiotic will effectively treat a certain bacterial infection. ***(Yes)***	111 (72.6%)	42 (22.2%)
Antibiotics treat infections from fungi, viruses, and bacteria ***(No)***	135 (88.2%)	18 (11.8%)
**Knowledge on antibiotic resistance**:		
Bacteria can become resistant to antibiotics. ***(Yes)***	151 (98.7%)	2 (1.3%)
The more antibiotics we use in the community, the higher the risk that resistance develops and spreads. ***(Yes)***	131 (85.6%)	22 (14.4%)
Non-compliance contributed to the development of antibiotic resistance. ***(Yes)***	123 (80.9%)	29 (19.1%)
Resistance can spread from animals to humans. ***(Yes)***	34 (22.2%)	119 (77.8%)
Resistance can spread from person to person. ***(Yes)***	69 (45.1%)	84 (54.9%)
Nowadays, antibiotic resistance is not a big problem in the world. ***(No)***	124 (81.1%)	29 (18.9%)
Taking antibiotics correctly may reduce the risk of antibiotic resistance ***(Yes)***	144 (94.1%)	9 (5.9%)
Antibiotics should be stopped immediately when the patient is clinically improved to reduce the risk of resistance ***(No)***	131 (85.6%)	22 (14.4%)
Physicians have a role to play in decreasing the prevalence of antibiotic resistance? ***(Yes)***	145 (94.8%)	8 (5.2%)
**Mean number of correct answers** *	12.4 ± 2.9	

* Correct answers from 17 questions.

**Fig 3:**
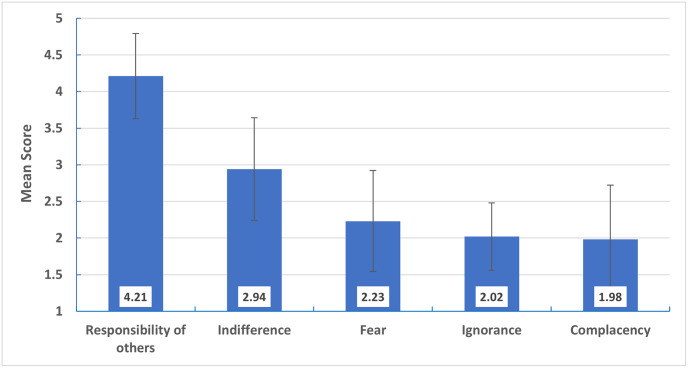
Mean attitudes score for inappropriate antibiotic prescribing. Error bars represent SD.

**Fig 4:**
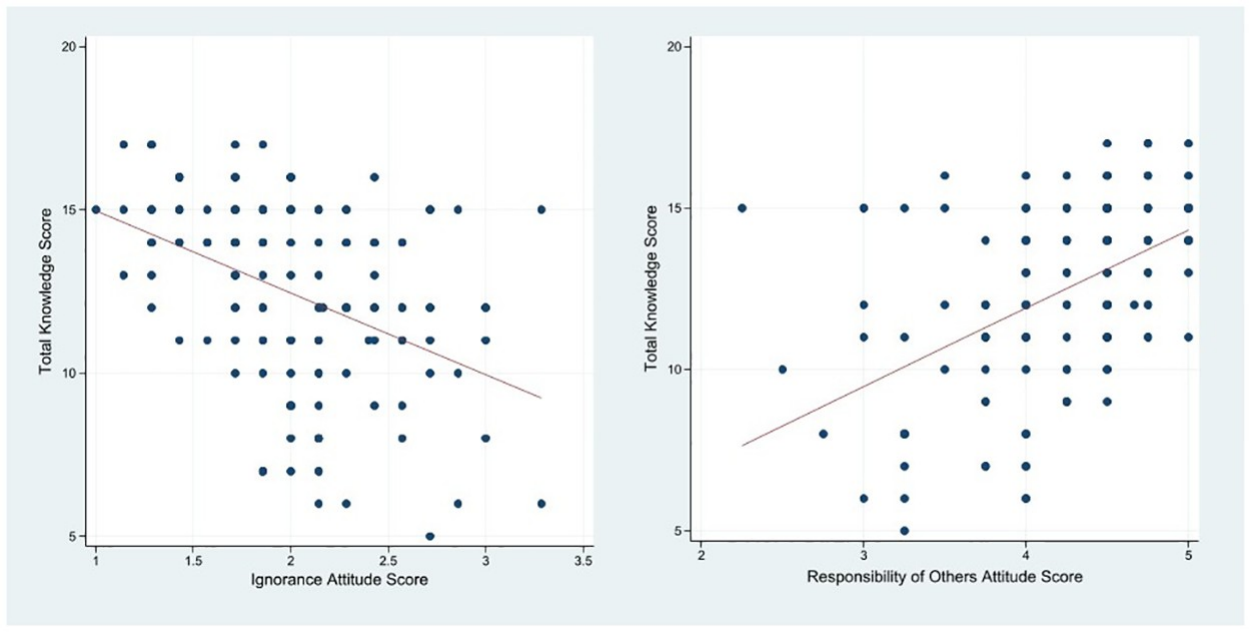
Correlation of total knowledge score with Ignorance and responsibility of others attitude scores.

Mean attitude scores for inappropriate antibiotic prescribing are illustrated in Fig 3, showing the responsibility for others has the highest score (4.21 ± 0.58), followed by the indifference score (2.94 ± 0.70). There was a significant negative correlation between knowledge score and ignorance attitude score (r = -0.428), p-value< 0.001) and significant positive correlation between knowledge scores and responsibility of others attitude score (r = 0.506, p-value< 0.001) (Fig 4).

**Fig 5:**
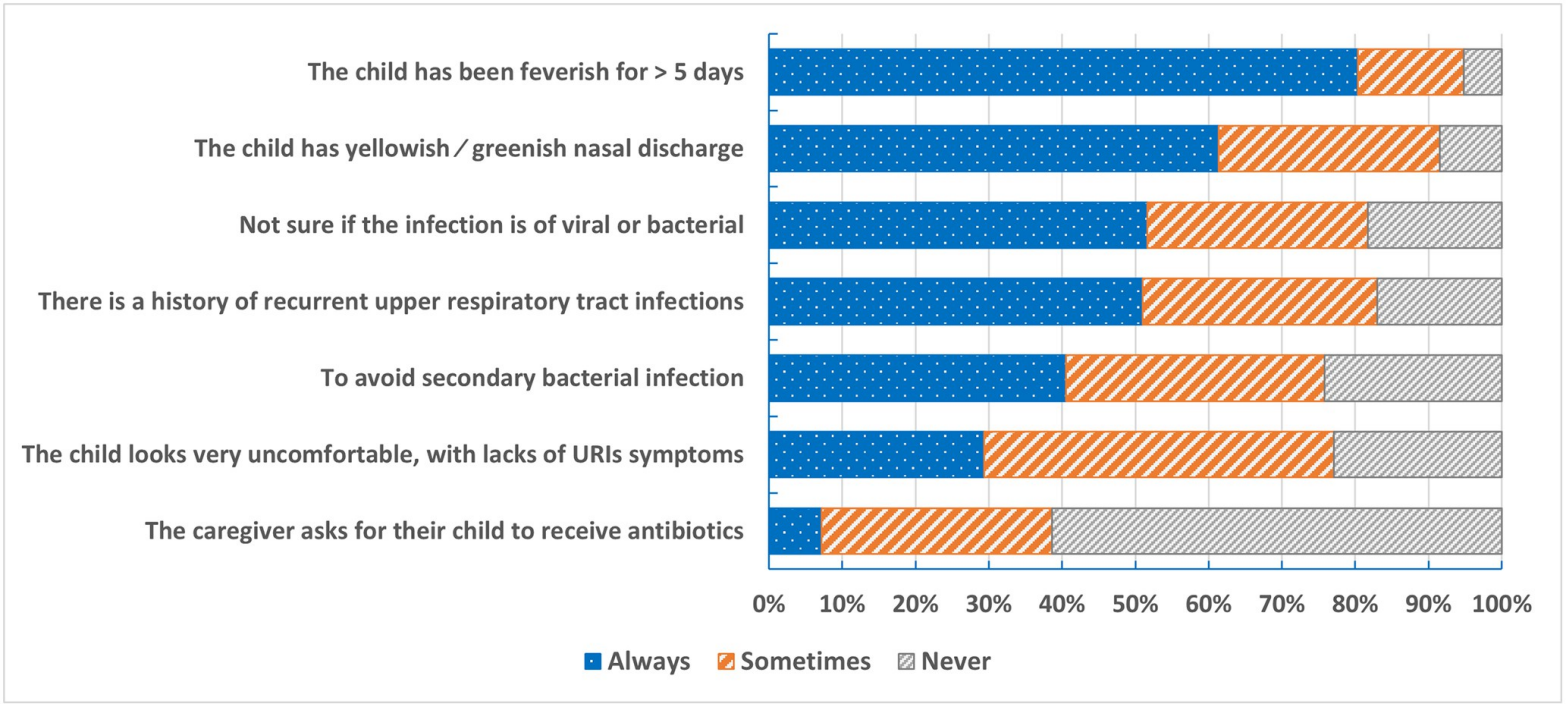
Prescribing practice in special conditions of URT infections.

Prescribing practice in special conditions of URT infections showed that 80% of participants always prescribe antibiotics if fever continues for more than five days, and 61.4% always prescribe antibiotics if the child has yellowish or greenish nasal discharge. On the other hand, 61.4% of participants do not prescribe antibiotics based on caregiver requests (Fig 5).

As each participant responded to four clinical vignettes, a total of 612 vignettes were assessed. Among them, 326 contained antibiotic prescriptions (53.3%). Appropriate antibiotic prescriptions represented only8.3% overall. By analysis of difference in knowledge scores between cases which prescribed inappropriate antibiotic (n = 299) and those with appropriate or no antibiotic prescriptions (n = 313), there was no statistically significant difference. The mean total knowledge score in inappropriate prescriptions was 12.3 ± 2.8 while in appropriate prescriptions and non-prescriptions group was 12.6 ± 2.9 (p-value = 0.170).

Further analysis of cases revealed that for case one, which is a viral infection and no need for antibiotics, 54 (35.3%) participants prescribed antibiotics. Most of the antibiotics prescribed in these cases were penicillin (70.4%), especially amoxicillin + clavulanic acid, which was the most frequent type used (39.5%). In 14.8% of cases, antibiotics were prescribed as an injection, and 3 participants mentioned that they would prescribe a combined antibiotic in this case. In this case, the mean duration of antibiotic use was 5.8 ± 1.4 days (Table 4).

**Table 4:** Participants’ responses to clinical vignettes.

	Case 1 N (%)	Case 2 N (%)	Case 3 N (%)	Case 4 N (%)
**Diagnosis**	**Viral URT infection**	**Common cold**	**Otitis media**	**Follicular tonsilitis**
Correct	141(92.2%%)	136 (88.9%)	140(91.5%)	147(96.0%)
Incorrect	9(5.9%)	9 (5.9%)	6(3.9%)	3(2.0%)
Uncertain	3(2.0%)	8(5.2%)	7(4.6%)	3(2.0%)
**Antibiotic prescription**				
Yes	54(35.3%)	11(7.2%)	112(73.2%)	149(97.4%)
No	99(64.7%)	142(92.8%)	41(26.8%)	4(2.6%)
**For Prescribed Cases**	**N = 54**	**N = 11**	**N = 112**	**N = 149**
**Antibiotic class and type**				
**Penicillin**	**38(70.4%)**	**7(63.6%)**	**94 (83.9%)**	**119 (79.9%)**
*Amoxicillin*	*9 (23*.*7%)*	*5 (71*.*4%)*	*34 (36*.*2%)*	*11 (9*.*2%)*
*Amoxicillin + clavulanic acid*	*15 (39*.*5%)*	*1 (14*.*3%)*	*35 (37*.*2%)*	*60 (50*.*4%)*
*Ampicillin sulbactam*	*4 (10*.*5%)*	*1*	*4 (4*.*3%)*	*38 (31*.*9%)*
*Amoxicillin + flucloxacillin*	*2 (5*.*3%)*	*0*	*7 (7*.*4%)*	*1 (0*.*84%)*
*Benzathine penicillin G*	*0*	*0*	*0*	*3 (2*.*5%)*
*Not specified penicillin*	*8 (21*.*1%)*	*0*	*14 (14*.*9%)*	*6 (5*.*0%)*
**Cephalosporins**	**4(7.4%)**	**1(9.1%)**	**17(15.2%)**	**24 (16.1%)**
*Ceftriaxone*	*1 (25*.*0%)*	*0*	*9 (52*.*9%)*	*11 (45*.*8%)*
*Other cephalosporins*	*3 (75*.*0%)*	*1 (100%)*	*8 (47*.*1%)*	*13 (54*.*2%)*
**Macrolides (azithromycin)**	**9(16.7%)**	**3(27.3%)**	**1(1.0%)**	**6(4.0%)**
**Not specified**	**3(5.6%)**	**0**	**0**	**0**
**Injectable antibiotic**	8(14.8%)	1(9.1%)	16(14.3%)	101(67.8%)
**Combined (more than 1) antibiotics**	3(5.6%)	2(18.2%)	11(9.8%)	49(32.9%)
**Duration of antibiotic use (days)**	5.8 ± 1.4	5.6 ± 1.7	6.8 ± 2.4	7.0 ± 2.4
**Rational antibiotic prescription**				
Appropriate prescription	0	0	15(13.4%)	12(8.0%)
Inappropriate prescription	54(100%)	11(100%)	97(86.6%)	137(92.0%)

Regarding the second case, a viral infection case (common cold), no antibiotic was needed; 11 (7.2%) participants were prescribed antibiotics. In this case, penicillin represented the most prescribed antibiotic class (63.6%), especially amoxicillin, which represented (71.4%) of all prescribed penicillin. Only one participant prescribed an injectable antibiotic, while 2 prescribed combined antibiotics. In this case, the mean duration of antibiotic use was 5.6 ± 1.7 days (Table 4).

The other two cases were bacterial infections where antibiotics were indicated. For the third case, otitis media, the most prescribed class was antibiotics and penicillin by 112 (73.2%). Amoxicillin and amoxicillin + clavulanic acid represented the most prescribed penicillin (36.2% and 37.2%, respectively). Also, cephalosporins were prescribed in 17 cases in which ceftriaxone represented 52.9%. Sixteen participants prescribed injectable antibiotics, representing 9.8%, while 11 (9.8%) prescribed combined antibiotics. In this case, the mean duration of antibiotic use was 6.8 ± 2.4 days. Only 15 (13.4%) were appropriate by assessing the appropriateness of prescribed antibiotics (Table 4).

Regarding the fourth case, follicular tonsilitis, 149 (97.4%) prescribed antibiotics, and penicillin was the most prescribed class. Amoxicillin + clavulanic acid represented the most prescribed penicillin (50.4%). Also, cephalosporins were prescribed in 24 cases, in which ceftriaxone represented 45.8% of them. Of the prescribed antibiotics, 101 (67.8%) were injectable, while 49 (32.9%) were combined antibiotics. In this case, the mean duration of antibiotic use was 7.0 ± 2.4 days. Only 12 (8.0%) were appropriate by assessing the appropriateness of prescribed antibiotics (Table 4).

## Discussion

Antimicrobial resistance (AMR) is one of the global threats nowadays. Increased antibiotic consumption in humans, animals, and agriculture has contributed directly to the spread of AMR [5]. In Egypt, a National Action Plan (NAP) for antimicrobial resistance was launched by the Ministry of Health and Population (MoHP) in collaboration with WHO in 2018 [7,32]. It is a nationwide multi-sectoral five-year plan (2018–2022) aiming to control and combat antimicrobial resistance through a One Health approach. The objectives of NAP are aligned with the global action plan based on national needs and priorities [32]. The current study acts toward achieving the first and second goals of this NAP, which focus on improving AMR’s awareness and understanding and promoting the rational use of antibiotics.

Overuse and consumption of antibiotics in healthcare are related to physicians’ prescribing behaviors as patients usually comply with their advice even if antibiotics are over-the-counter (OTC) drugs in the country. However, the prescription by the physician is still the main driver for using it [10]. Antibiotic prescribing practices rely on multiple factors, namely, the intrinsic factors such as knowledge and attitude of prescribers and external factors such as patients and the institutional environment [33]. knowledge and attitudes are the core of intrinsic determinants of antibiotic prescribing practices. Knowledge can influence prescribing behaviors directly or indirectly through influencing attitudes. The knowledge and attitudes of prescribers can be shaped by many sociodemographic characteristics, such as gender, qualifications, clinical expertise, continuing education, and years of practices [24]. Furthermore, when physicians prescribe medicines, they are likely to take considerations of many external factors apart from relevant clinical standards and guidelines. Pressures from patients, peers, employers, governments, and the public may affect antibiotic prescribing decisions. These factors can exert impacts on prescribing practices directly or indirectly through influencing attitudes [24,34].

Most of the recruited physicians in this study were less than 35 years old. This was expected as this study aimed to assess antibiotic prescriptions in GPs and physicians who have not yet specialized or hold a degree. In Egypt’s health system, most medical school graduates enroll in postgraduate studies or fellowship programs to become specialized directly after graduation. This also explains this study’s small proportion of GPs [35]. Moreover, as the study looked for antibiotic prescribing in pediatric cases, most participants were pediatric residents.

Previous studies demonstrated that continuous medical education improves antibiotic prescribing behaviors [36]. In the current study, only 28% of participants attended a CME event last year addressing antibiotic use and resistance. Another crucial training also contributed to improving antibiotic prescriptions is the integrated management of childhood illness (IMCI) [37]. In the current study, nearly half of the participants attended IMCI training, and 43% reported using IMCI guidelines in their practice. In Egypt, the IMCI training program has been provided as pre-employment training for MoHP physicians since 2002 [38].

Based on the Teixeira Antibiotic Prescribing Behavioral Model (TAPBM), knowledge and attitudes toward antibiotic use and resistance significantly impact prescribing practice [33]. In the present study, participants showed sufficient knowledge about antibiotic use and resistance. The knowledge result is consistent with the Egyptian national survey conducted by El-Sokarry et al., in which participating physicians showed good knowledge [19]. Furthermore, the most incorrectly reported information was related to the transmission of resistant organisms from animal to human and from person to person. Also, this was reported in El-Sokkary et al.’s survey, in which more than half of the physicians did not know that the resistant organism could be transmitted from person to person [19]. Modifications added to medical curriculum in the last couple of years may have affected the medical school graduates’ knowledge. Although of good knowledge scores, high percentage of inappropriate prescriptions was reported in the current study that may be explained by that, curriculum changes mainly focused on theoretical knowledge “Passive learning” and not integrated in active learning of case management [39,40].

Regarding participant attitudes, the responsibility of others (i.e., other physicians, patients, or other healthcare providers) was the highest attitude score, consistent with the result of a systematic review by Rodrigues et al., in which 35 studies for physician antibiotic prescribing behavior were reviewed. In this review, the responsibility of others was described as influencing antibiotic prescribing in six papers [24]. Moreover, in the current study, the indifference score was higher than the complacency, fear, and ignorance scores, which may be linked to inappropriate antibiotic prescribing behavior. This is consistent with Rodrigues et al.’s review, as they reported a direct relationship between high indifference scores and inappropriate antibiotic prescriptions [24]. Although ignorance of antibiotic resistance, fear of complications, and complacency with patients’ expectations and pressure were reported in many studies as essential factors for inappropriate antibiotic prescribing behavior, participants in the current study had low scores for those attitudes [24,34,41]. The low complacency score was consistent with participants’ reports about the factors affecting their antibiotic prescribing and cases of acute URTIs in which patients’ expectations were the most negligible factor. Moreover, most of them never prescribe antibiotics when the caregiver asks for them.

Many studies report patient-related factors affecting antibiotic prescribing behavior. Physicians may feel that specific clinical symptoms frequently connected with prescriptions suggest a bacterial infection or a more severe illness [42]. As reported in many studies for GPs’ antibiotic prescriptions in cases of acute RTIs, the following respiratory symptoms are associated with increased odds of antibiotic prescriptions: fever> 38°C, productive cough, otalgia, bulging tympanic membrane, otorrhea, and tender cervical nodes [43–46]. In the current study, clinical assessment and reported symptoms were the factors that most affected the antibiotic prescribing decision. Furthermore, more than half of the participants always prescribed antibiotics in cases of fever lasting longer than five days or nasal discharge that is greenish or yellowish, which is consistent with the findings from prior studies, and was not justified according to the guidelines [47,48].

Clinical vignettes are an effective tool to explore antibiotic prescribing patterns for different cases and have been used previously in many studies [49]. In the current study, participants prescribed antibiotics for more than half of the cases (53%), and most of the prescriptions were inappropriate (91.7%). A study conducted in Minya district in Egypt revealed that 82% of pediatric cases were prescribed antibiotics, and 80% were not [50]. These results are consistent with many studies from developing countries; as in the Chinese study, antibiotics were prescribed for 59% of pediatrics URTIs cases, and 91.3% were inappropriate [51]. While in India, in PHC facilities, 85% of patients had antibiotic prescriptions, and nearly 61.0% of prescribed antibiotics for children were inappropriate [52]. In Yemen, a study investigated antibiotic prescription patterns at outpatient departments (OPDs) at four hospitals in Aden. The study revealed that the percentage of prescriptions involving antibiotics was 84%. Upper respiratory tract infections represent 37% of cases that received antibiotics [53]. In Saudi Arabia, a study conducted in Makkah PHC centers revealed that antibiotics were prescribed in 56.7% of the cases, and most of these cases were for simple common cold [54].

Among the inappropriate prescriptions in the current study, antibiotics were prescribed for 7% of common cold cases and 35% of viral URT infection cases with a sore throat. This percentage is low compared to other studies conducted in Egypt by examining prescriptions in which antibiotics were prescribed for 53.3% of common cold cases and 96% of viral pharyngitis cases. This difference may be attributed to using case vignettes rather than being like countering real patients, and responses may represent socially desirable answers rather than actual antibiotic prescribing practice [49]. However, the most prescribed type of antibiotic was penicillin, especially amoxicillin and amoxicillin + clavulanic acid, and this is consistent with other studies which investigated the types of antibiotics used in acute URTIs [54–57]. The type of prescribed antibiotics in such cases should be considered as in cases of vial tonsilitis caused by infection mononucleosis (IM); the use of aminopenicillins as amoxicillin is associated with development of skin rashes [58]. The exact mechanism behind them is unclear. It is not well explained yet, whether a true allergic drug reaction or virus-dependent rash. The rash may be due to the viral infection itself, the incidence of skin eruption development in acute IM is 4.2–13% without drug intake, but often these patients are put on antibiotics, frequently amoxicillin, and the rash appears a few days after the initiation of the antibiotic therapy [59,60]. Following amoxicillin intake within acute IM the incidence of skin reactions ranges between 27.8% and 69% in adults while in children, morbilliform skin eruptions nearly always develop following amoxicillin intake within acute IM [60,61].

In the present study, the antibiotic prescriptions for bacterial cases in which antibiotics are indicated are consistent with other studies. However, the percentage of inappropriate prescriptions was high due to the wrong choice, duration of antibiotics, or use of injectable or combined antibiotics. For otitis media (OM) cases, 73% of participants prescribed antibiotics, and non-prescribing antibiotics here cannot be considered inappropriate as some guidelines recommend delayed prescription or even nonprescription if not complicated. Among those prescriptions, nearly 87% were inappropriate, and most were due to inappropriate duration, as the recommended duration for OM treatment is five days, while in this study, the mean duration was 6.8 ± 2.4.

On the other hand, 97% of follicular tonsilitis cases had an antibiotic prescription, and 92% were inappropriate. Hence, the inappropriate type and duration together composed most of the inappropriateness. For those cases, the most prescribed antibiotic was amoxicillin + clavulanic acid (40%). However, guidelines do not recommend the antibiotic of choice as amoxicillin and/or penicillin V, except for penicillin allergy [26,28]. Also, injectable and combined antibiotics were high in those cases, which exposed children to more complications. Non-adherence to antibiotic guidelines is a common problem in cases of acute bacterial tonsilitis. In our literature search, we found many studies that assessed the proportion of patients not receiving the first-choice antibiotic treatment for bacterial tonsilitis. In these investigations, the non-compliance rate with guidelines ranged from 27.0% to 63.0% among children [62–65]. However, no study was conducted to deeply explore why there is a high rate of non-adherence to guidelines in those cases.

Although this study is a descriptive and aiming to explore the current practice, but it is obvious that, there is a need to introduce corrective actions to control antibiotic over prescription in such cases. Interventions that attempt to improve antibiotic prescribing tend to be more successful when they combine doctor, patient and public education and when the design is multi-faceted [66–69]. A systematic review that focused on antibiotic use for RTIs, found that multiple interventions that contained at least educational materials for doctors were most effective [70]. In current study the main problem was in practice and adherence to guidelines, so we need to adopt interventions that act toward monitoring and improvement of physicians’ practical knowledge and action. One of suggested interventions is medical audit and feedback as in the Audit Project, Swedish general practitioners (GPs) recorded their consultations at two time points and received feedback in between. The medical audit resulted in a 10% decrease in antibiotic prescription within the intervention group [71]. Providing individual prescribing feedback has also been shown to lead to improvements in guideline adherence [72]. Other recommended corrective tool is educational sessions for physicians; a review of interventions targeting doctors to improve antibiotic use for RTIs showed that educational sessions, appear to be more effective than audit and feedback [73]. Educational sessions can include: information on the core principles of rational antibiotic use, introduction to new tools (guidelines for example), diagnostic skills training and sessions on patient communication [74,75]. A follow-up study for educational intervention has been conducted in Netherlands and reported that, sessions incorporating these subject areas can lead to sustained improvement in antibiotic use that can last longer than two to four years [76]. In designing an educational program for this problem, it is advisable to use WHO competency framework for health workers’ education and training on antimicrobial resistance. This WHO competency framework for education on AMR is strategic and timely, given the widespread perception among health workers of insufficient knowledge and expertise on the topic, resulting in inappropriate antimicrobial prescription and use practices [77]. Those competences can be delivered either in pre-service or in-service trainings. The framework containing core and additional AMR competencies, which have been organized across four domain areas. The domain areas include foundations that build awareness of antimicrobial resistance, appropriate use of antimicrobial agents, infection prevention and control (IPC), and diagnostic stewardship and surveillance. In each domain, the framework provides knowledge, skills and attitudes that should be acquired by healthcare workers either prescribers or non-prescribers and also by public health officers and health services managers [77].

Our study relied on physician recall and self-reported practices, which has significant limitations. We did not evaluate the relationship of attitudes to actual antimicrobial prescribing. Therefore, there may have been information bias because of a tendency for respondents to provide answers they believed the researchers wanted to hear, resulting in an underestimation of the prevalence of improper attitudes and prescription behaviors. Other factors influencing physician attitudes regarding antibiotic prescribing, such as pharmaceutical promotion, restricted consultation, dispensing times, and financial incentives, were not investigated. The research was conducted in a single Egyptian district, limiting its generalizability. The four clinical vignettes used to evaluate antibiotic prescriptions were limited to a few features, not actual patients. Furthermore, they did not permit ordering further point-of-care diagnostic testing as would have been appropriate before administering antibiotics.

Moreover, we did not measure antibiotic consumption and prescription audit to determine the appropriate use of antibiotics and the efficacy of interventions. Although these limitations, this study is the first evaluation of knowledge, attitudes, and practices reported regarding antibiotic prescriptions for treating acute URTIs in children by pediatricians in Assiut, Egypt. The results of this survey can be used to improve education, antimicrobial surveillance, and antibiotic prescribing patterns among physicians in our setting.

## Conclusions

Physicians dealing with acute URTIs in outpatients’ clinics in Assiut district have good knowledge about antibiotic use and resistance and demonstrate a good attitude toward appropriate antibiotic use. However, the percentage of inappropriate prescriptions for clinical vignettes is high. More research is required to determine the causes of improper antibiotic prescribing and non-compliance with guidelines. Also, it is crucial to set up a national antibiotic stewardship program to improve antibiotic prescribing and contain antimicrobial resistance problems.

## Abbreviations

AMR: Antimicrobial resistance
AWaRe: Access, Watch and Reserve guidelines
CME: Continuous Medical Education
ENT: Ear, Nose and Throat
FAO: Food and Agriculture Organization
GAP: Global Action Plan
GPs: General Practitioners
IM: Intra-muscular
IMCI: Integrated Management of Childhood Illness
MoHP: Ministry of Health and Population
NAP: National Action Plan
OM: Otitis Media
OTC: Over the Counter
PHC: Primary Health Care
SD: Standard Deviation
STROBE: Strengthening the Reporting of Observational Studies in Epidemiology
TAPBM: Teixeira Antibiotic Prescribing Behavioral Model
URIs: Upper Respiratory Tract infections
WHO: World Health Organization
WHO’s-INRUD: the World Health Organization’s International Network on the Rational Use of Drugs program
WOAH: World Organization for Animal Health

## References

[1] Uddin TM, Chakraborty AJ, Khusro A, Zidan BRM, Mitra S, Bin Emran T, et al. Antibiotic resistance in microbes: History, mechanisms, therapeutic strategies and future prospects. J Infect Public Health 2021;14:1750–66. doi: 10.1016/j.jiph.2021.10.020 34756812

[2] da Cunha BR, Fonseca LP, Calado CRC. Antibiotic Discovery: Where Have We Come from, Where Do We Go? Antibiotics 2019;8. doi: 10.3390/ANTIBIOTICS8020045 31022923 PMC6627412

[3] Munita JM, Arias CA. Mechanisms of Antibiotic Resistance. Virulence Mech Bact Pathog 2016:481–511. doi: 10.1128/microbiolspec.VMBF-0016-2015 27227291 PMC4888801

[4] MacGowan A, Macnaughton E. Antibiotic resistance. Med (United Kingdom) 2017;45:622–8. doi: 10.1016/j.mpmed.2017.07.006

[5] WHO. Antibiotic resistance-Fact sheets 2018. https://www.who.int/news-room/fact-sheets/detail/antibiotic-resistance.

[6] Murray CJ, Ikuta KS, Sharara F, Swetschinski L, Robles Aguilar G, Gray A, et al. Global burden of bacterial antimicrobial resistance in 2019: a systematic analysis. Lancet 2022;399:629–55. doi: 10.1016/S0140-6736(21)02724-0 35065702 PMC8841637

[7] WHO. Global action plan on antimicrobial resistance. WHO Press 2015:1–28. ISBN 978 92 4 150976 3.

[8] WHO. Antimicrobial resistance. Global Report on Surveillance. Bull World Health Organ 2014;61:383–94. doi: 10.1007/s13312-014-0374-3 24736901

[9] Austin DJ, Kristinsson KG, Anderson RM. The relationship between the volume of antimicrobial consumption in human communities and the frequency of resistance. Proc Natl Acad Sci 1999. doi: 10.1073/pnas.96.3.1152 9927709 PMC15366

[10] Tamma PD, Cosgrove SE. Let the games begin: The race to optimise antibiotic use. Lancet Infect Dis 2014;14:667–8. doi: 10.1016/S1473-3099(14)70809-6 25022437

[11] Lange K, Buerger M, Stallmach A, Bruns T. Effects of Antibiotics on Gut Microbiota. Dig Dis 2016;34:260–8. doi: 10.1159/000443360 27028893

[12] Shaw SY, Blanchard JF, Bernstein CN. Association Between the Use of Antibiotics in the First Year of Life and Pediatric Inflammatory Bowel Disease. Am J Gastroenterol 2010. doi: 10.1038/ajg.2010.398 20940708

[13] Risnes KR, Belanger K, Murk W, Bracken MB. Antibiotic exposure by 6 months and asthma and allergy at 6 years: Findings in a cohort of 1,401 US children. Am J Epidemiol 2011. doi: 10.1093/aje/kwq400 21190986 PMC3105273

[14] Cox LM, Blaser MJ. Antibiotics in early life and obesity. Nat Rev Endocrinol 2015. doi: 10.1038/nrendo.2014.210 25488483 PMC4487629

[15] Neuman H, Forsythe P, Uzan A, Avni O, Koren O. Antibiotics in early life: dysbiosis and the damage done. FEMS Microbiol Rev 2018;42:489–99. doi: 10.1093/femsre/fuy018 29945240

[16] Hersh AL, Shapiro DJ, Pavia AT, Shah SS. Antibiotic Prescribing in Ambulatory Pediatrics in the United States. Pediatrics 2011. doi: 10.1542/peds.2011-1337 22065263

[17] Jacobs MR, Johnson CE. Macrolide resistance: An increasing concern for treatment failure in children. Pediatr Infect Dis J 2003;22. doi: 10.1097/00006454-200308001-00004 14566999

[18] McCaig LF, Besser RE, Hughes JM. Trends in antimicrobial prescribing rates for children and adolescents. J Am Med Assoc 2002. doi: 10.1001/jama.287.23.3096 12069672

[19] El-Sokkary R, Kishk R, El-Din SM, Nemr N, Mahrous N, Alfishawy M, et al. Antibiotic use and resistance among prescribers: Current status of knowledge, attitude, and practice in Egypt. Infect Drug Resist 2021;14:1209–18. doi: 10.2147/IDR.S299453 33790591 PMC8007586

[20] Dooling KL, Kandeel A, Hicks LA, El-Shoubary W, Fawzi K, Kandeel Y, et al. Understanding antibiotic use in Minya district, Egypt: Physician and pharmacist prescribing and the factors influencing their practices. Antibiotics 2014;3:233–43. doi: 10.3390/antibiotics3020233 27025746 PMC4790392

[21] Von Elm E, Altman DG, Egger M, Pocock SJ, Gøtzsche PC, Vandenbroucke JP. The Strengthening the Reporting of Observational Studies in Epidemiology (STROBE) Statement: Guidelines for Reporting Observational Studies. PLOS Med 2007;4:e296. doi: 10.1371/journal.pmed.0040296 17941714 PMC2020495

[22] CAPMAS. Assiut Governorate Population Statistics. 2018.

[23] Rodrigues AT, Ferreira M, Piñeiro-Lamas M, Falcão A, Figueiras A, Herdeiro MT. Determinants of physician antibiotic prescribing behavior: A 3 year cohort study in Portugal. Curr Med Res Opin 2016;32:949–57. doi: 10.1185/03007995.2016.1154520 26878083

[24] Teixeira Rodrigues A, Roque F, Falcão A, Figueiras A, Herdeiro MT. Understanding physician antibiotic prescribing behaviour: A systematic review of qualitative studies. Int J Antimicrob Agents 2013;41:203–12. doi: 10.1016/j.ijantimicag.2012.09.003 23127482

[25] Liu CC, Liu CC, Wang D, Zhang X. Knowledge, attitudes and intentions to prescribe antibiotics: A structural equation modeling study of primary care institutions in Hubei, China. Int J Environ Res Public Health 2019;16:2385. doi: 10.3390/ijerph16132385 31284381 PMC6651188

[26] WHO. Access-Watch-Reserve The AWaRe categorization of antibiotics on the WHO Model List of Essential Medicines 2017.

[27] Committee IC. Assiut University Hospitals Antibiotic Guidline 2016.

[28] Bradley JS, Barnett ED, Cantey JB, Kimberlin DW, Palumbo PE, Sauberan J, et al. Nelson’s Pediatric Antimicrobial Therapy 2021. 27th ed. American Academy of Pediatrics Publishing Staff; 2021.

[29] WHO. WHO | WHO Global Strategy for Containment of Antimicrobial Resistance. World Health Organization; 2016.

[30] Yaghmaie F. Content validity and its estimation. J Med Educ 2003;3:7–25.

[31] Alumran A, Hou XY, Hurst C. Validity and reliability of instruments designed to measure factors influencing the overuse of antibiotics. J Infect Public Health 2012;5:221–32. doi: 10.1016/j.jiph.2012.03.003 22632596

[32] WHO. Egypt National Action Plan For Antimicrobial Resistance 2018–2022. WHO office in Egypt; 2018.

[33] Thakolkaran N, Shetty Av, D′Souza NR, Shetty A. Antibiotic prescribing knowledge, attitudes, and practice among physicians in teaching hospitals in South India. J Fam Med Prim Care 2017;6:526. doi: 10.4103/2249-4863.222057 29417002 PMC5787949

[34] Liu C, Liu C, Wang D, Zhang X. Intrinsic and external determinants of antibiotic prescribing: A multi-level path analysis of primary care prescriptions in Hubei, China. Antimicrob Resist Infect Control 2019;8:1–12. doi: 10.1186/S13756-019-0592-5/FIGURES/2 31406571 PMC6686458

[35] Abdelaziz A, Kassab SE, Abdelnasser A, Hosny S. Medical Education in Egypt: Historical Background, Current Status, and Challenges. Heal Prof Educ 2018;4:236–44. doi: 10.1016/J.HPE.2017.12.007

[36] Om C, Vlieghe E, McLaughlin JC, Daily F, McLaws ML. Antibiotic prescribing practices: A national survey of Cambodian physicians. Am J Infect Control 2016;44:1144–8. doi: 10.1016/j.ajic.2016.03.062 27324610

[37] Carai S, Kuttumuratova A, Boderscova L, Khachatryan H, Lejnev I, Monolbaev K, et al. The integrated management of childhood illness (IMCI) and its potential to reduce the misuse of antibiotics. J Glob Health 2021;11:04029. doi: 10.7189/jogh.11.04029 34055327 PMC8141328

[38] WHO. Implementation of IMCI in Egypt 2014. http://www.emro.who.int/child-health/strategy-implementation/implementation-of-imci-in-egypt.html (accessed June 14, 2022).

[39] Assar A, Abdelraoof MI, Abdel-Maboud M, Shaker KH, Menshawy A, Swelam AH, et al. Knowledge, attitudes, and practices of Egypt’s future physicians towards antimicrobial resistance (KAP-AMR study): a multicenter cross-sectional study. Environ Sci Pollut Res 2020;27:21292–8. doi: 10.1007/s11356-020-08534-5 32270452

[40] Lee CR, Lee JH, Kang LW, Jeong BC, Lee SH. Educational effectiveness, target, and content for prudent antibiotic use. Biomed Res Int 2015;2015. doi: 10.1155/2015/214021 25945327 PMC4402196

[41] Gonzalez-Gonzalez C, López-Vázquez P, Vázquez-Lago JM, Piñeiro-Lamas M, Herdeiro MT, Arzamendi PC, et al. Effect of physicians’ attitudes and knowledge on the quality of antibiotic prescription: A cohort study. PLoS One 2015;10:e0141820. doi: 10.1371/journal.pone.0141820 26509966 PMC4624842

[42] McKay R, Mah A, Law MR, McGrail K, Patrick DM. Systematic Review of Factors Associated with Antibiotic Prescribing for Respiratory Tract Infections. Antimicrob Agents Chemother 2016;60:4106–18. doi: 10.1128/AAC.00209-16 27139474 PMC4914667

[43] Saliba-Gustafsson EA, Dunberger Hampton A, Zarb P, Orsini N, Borg MA, Stålsby Lundborg C. Factors associated with antibiotic prescribing in patients with acute respiratory tract complaints in Malta: a 1-year repeated cross-sectional surveillance study. BMJ Open 2019;9:e032704. doi: 10.1136/bmjopen-2019-032704 31857311 PMC6937012

[44] Moro ML, Marchi M, Gagliotti C, Di Mario S, Resi D. Why do paediatricians prescribe antibiotics? Results of an Italian regional project. BMC Pediatr 2009;9:69. doi: 10.1186/1471-2431-9-69 19895678 PMC2777860

[45] Manne M, Deshpande A, Hu B, Patel A, Taksler GB, Misra-Hebert AD, et al. Provider variation in antibiotic prescribing and outcomes of respiratory tract infections. South Med J 2018;111:235–42. doi: 10.14423/SMJ.0000000000000795 29719037 PMC8564887

[46] Alrafiaah AS, Alqarny MH, Alkubedan HY, AlQueflie S, Omair A. Are the Saudi parents aware of antibiotic role in upper respiratory tract infections in children? J Infect Public Health 2017;10:579–85. doi: 10.1016/j.jiph.2017.01.023 28283368

[47] O’Connor R, O’Doherty J, O’Regan A, Dunne C. Antibiotic use for acute respiratory tract infections (ARTI) in primary care; what factors affect prescribing and why is it important? A narrative review. Ir J Med Sci 2018;187:969–86. doi: 10.1007/s11845-018-1774-5 29532292 PMC6209023

[48] Ye D, Yan K, Zhang H, Liu S, Yang C, Jiang M, et al. A survey of knowledge, attitudes and practices concerning antibiotic prescription for upper respiratory tract infections among pediatricians in 2018 in Shaanxi Province, China. Expert Rev Anti Infect Ther 2020;18:927–36. doi: 10.1080/14787210.2020.1761789 32338547

[49] Lucet J-C, Nicolas-Chanoine M-H, Lefort A, Roy C, Diamantis S, Papy E, et al. Do Case Vignettes Accurately Reflect Antibiotic Prescription? Infect Control Hosp Epidemiol 2011;32:1003–9. doi: 10.1086/661914 21931251

[50] Kandeel A, Palms DL, Afifi S, Kandeel Y, Etman A, Hicks LA, et al. An educational intervention to promote appropriate antibiotic use for acute respiratory infections in a district in Egypt- pilot study. BMC Public Health 2019;19:498. doi: 10.1186/s12889-019-6779-0 32326918 PMC6696705

[51] Chang Y, Chusri S, Sangthong R, McNeil E, Hu J, Du W, et al. Clinical pattern of antibiotic overuse and misuse in primary healthcare hospitals in the southwest of China. PLoS One 2018;14:e0214779. doi: 10.1371/journal.pone.0214779 31242185 PMC6594576

[52] Basu S, Chatterjee M, Chandra PK, Basu S. Antibiotic misuse in children by the primary care physicians an Indian experience. Niger J Clin Pract 2008;11:52–7. 18689140

[53] Alshakka M, Said K, Babakri M, Ansari M, Aldhubhani A, Azmi Hassali M, et al. A Study on Antibiotics Prescribing Pattern at Outpatient Department in Four Hospitals in Aden-Yemen. J Pharm Pract Community Med 2016;2:88–93. doi: 10.5530/jppcm.2016.3.5

[54] Shaheen MH, Siddiqui MI, Jokhdar HA, Hassan-Hussein A, Garout MA, Hafiz SM, et al. Prescribing Patterns for Acute Respiratory Infections in Children in Primary Health Care Centers, Makkah Al Mukarramah, Saudi Arabia. J Epidemiol Glob Health 2018;8:149. doi: 10.2991/j.jegh.2017.10.007 30864756 PMC7377574

[55] Marc C, Vrignaud B, Levieux K, Robine A, Guen CG Le, Launay E. Inappropriate prescription of antibiotics in pediatric practice: Analysis of the prescriptions in primary care. J Child Heal Care 2016;20:530–6. doi: 10.1177/1367493516643421 27091956

[56] Keohavong B, Vonglokham M, Phoummalaysith B, Louangpradith V, Inthaphatha S, Kariya T, et al. Antibiotic prescription for under-fives with common cold or upper respiratory tract infection in Savannakhet Province, Lao PDR. Trop Med Health 2019;47:16. doi: 10.1186/s41182-019-0143-z 30858755 PMC6394019

[57] Alkaff RN, Kamigaki T, Saito M, Ariyanti F, Iriani DU, Oshitani H. Use of antibiotics for common illnesses among children aged under 5 years in a rural community in indonesia: A cross-sectional study. Trop Med Health 2019;47:1–9. doi: 10.1186/s41182-019-0173-6 31360099 PMC6639925

[58] Van Der Linden PD, Van Der Lei J, Vlug AE, Stricker BHC. Skin reactions to antibacterial agents in general practice. J Clin Epidemiol 1998;51:703–8. doi: 10.1016/s0895-4356(98)00041-9 9743319

[59] Chovel-Sella A, Ben Tov A, Lahav E, Mor O, Rudich H, Paret G, et al. Incidence of rash after amoxicillin treatment in children with infectious mononucleosis. Pediatrics 2013;131. doi: 10.1542/peds.2012-1575 23589810

[60] Ónodi-Nagy K, Kinyó Á, Meszes A, Garaczi E, Kemény L, Bata-Csörgo Z. Amoxicillin rash in patients with infectious mononucleosis: evidence of true drug sensitization. Allergy Asthma Clin Immunol 2015;11. doi: 10.1186/1710-1492-11-1 25784943 PMC4362637

[61] Renn CN, Straff W, Dorfmüller A, Al-Masaoudi T, Merk HF, Sachs B. Amoxicillin-induced exanthema in young adults with infectious mononucleosis: Demonstration of drug-specific lymphocyte reactivity. Br J Dermatol 2002;147:1166–70. doi: 10.1046/j.1365-2133.2002.05021.x 12452866

[62] Murphy M, Bradley CP, Byrne S. Antibiotic prescribing in primary care, adherence to guidelines and unnecessary prescribing—an Irish perspective. BMC Fam Pract 2012;13. doi: 10.1186/1471-2296-13-43 22640399 PMC3430589

[63] Williams MR, Greene G, Naik G, Hughes K, Butler CC, Hay AD. Antibiotic prescribing quality for children in primary care: an observational study. Br J Gen Pract 2018;68:e90–6. doi: 10.3399/bjgp18X694409 29335323 PMC5774968

[64] Rico-Ferreira P, Palazón-Bru A, Calvo-Pérez M, Gil-Guillén VF. Nonadherence to guidelines for prescribing antibiotic therapy to patients with tonsillitis or pharyngotonsillitis: a cross-sectional study. Curr Med Res Opin 2015;31:1319–22. doi: 10.1185/03007995.2015.1041896 25876462

[65] Tell D, Engström S, Mölstad S. Adherence to guidelines on antibiotic treatment for respiratory tract infections in various categories of physicians: A retrospective cross-sectional study of data from electronic patient records. BMJ Open 2015;5. doi: 10.1136/bmjopen-2015-008096 26179648 PMC4513445

[66] Bjerrum L, Munck A, Gahrn-Hansen B, Hansen MP, Jarbol DE, Cordoba G, et al. Health Alliance for prudent antibiotic prescribing in patients with respiratory tract infections (HAPPY AUDIT) -impact of a non-randomised multifaceted intervention programme. BMC Fam Pract 2011 121 2011;12:1–8. doi: 10.1186/1471-2296-12-52 21689406 PMC3146837

[67] Butler CC, Rollnick S, Pill R, Maggs-Rapport F, Stott N. Understanding the culture of prescribing: qualitative study of general practitioners’ and patients’ perceptions of antibiotics for sore throats. BMJ 1998;317:637–42. doi: 10.1136/bmj.317.7159.637 9727992 PMC28658

[68] Arnold SR, Straus SE. Interventions to improve antibiotic prescribing practices in ambulatory care. Cochrane Database Syst Rev 2005;2005. doi: 10.1002/14651858.CD003539.pub2 16235325 PMC7003679

[69] Wilkinson A, Ebata A, Macgregor H. Interventions to reduce antibiotic prescribing in LMICs: A scoping review of evidence from human and animal health systems. Antibiotics 2019;8:2. doi: 10.3390/antibiotics8010002 30583566 PMC6466578

[70] Wei X, Zhang Z, Hicks JP, Walley JD, King R, Newell JN, et al. Long-term outcomes of an educational intervention to reduce antibiotic prescribing for childhood upper respiratory tract infections in rural China: Follow-up of a cluster-randomised controlled trial. PLoS Med 2019. doi: 10.1371/journal.pmed.1002733 30721234 PMC6363140

[71] Melander E, Björgell A, Björgell P, Ovhed I, Mölstad S. Medical audit changes physicians’ prescribing of antibiotics for respiratory tract infections. Scand J Prim Health Care 1999;17:180–4. doi: 10.1080/028134399750002610 10555249

[72] Hürlimann D, Limacher A, Schabel M, Zanetti G, Berger C, Mühlemann K, et al. Improvement of antibiotic prescription in outpatient care: a cluster-randomized intervention study using a sentinel surveillance network of physicians. J Antimicrob Chemother 2015;70:602–8. doi: 10.1093/jac/dku394 25326088

[73] Van Der Velden AW, Pijpers EJ, Kuyvenhoven MM, Tonkin-Crine SKG, Little P, Verheij TJM. Effectiveness of physician-targeted interventions to improve antibiotic use for respiratory tract infections. Br J Gen Pract 2012;62:e801. doi: 10.3399/bjgp12X659268 23211259 PMC3505412

[74] Dyar OJ, Beović B, Vlahović-Palčevski V, Verheij T, Pulcini C. How can we improve antibiotic prescribing in primary care? Expert Rev Anti Infect Ther 2016;14:403–13. doi: 10.1586/14787210.2016.1151353 26853235

[75] Little P, Stuart B, Francis N, Douglas E, Tonkin-Crine S, Anthierens S, et al. Effects of internet-based training on antibiotic prescribing rates for acute respiratory-tract infections: a multinational, cluster, randomised, factorial, controlled trial. Lancet (London, England) 2013;382:1175–82. doi: 10.1016/S0140-6736(13)60994-0 23915885 PMC3807804

[76] Cals JWL, de Bock L, Beckers PJHW, Francis NA, Hopstaken RM, Hood K, et al. Enhanced communication skills and C-reactive protein point-of-care testing for respiratory tract infection: 3.5-year follow-up of a cluster randomized trial. Ann Fam Med 2013;11:157–64. doi: 10.1370/afm.1477 23508603 PMC3601394

[77] World Health Organization. WHO competency framework for health workers’ education and training on antimicrobial resistance. World Heal Organ 2018.

